# A Retrospective Analysis of the Effectiveness of Fractional CO₂ Laser Therapy in Treating Linear Scars: Investigating the Ideal Timing for Intervention

**DOI:** 10.1111/jocd.70019

**Published:** 2025-03-04

**Authors:** Ganlin Gu, Liyue Ji, Xiaonan Qiu, Jing Zhang

**Affiliations:** ^1^ Department of Dermatology, The Eighth Affiliated Hospital Sun Yat‐sen University Shenzhen China; ^2^ Department of Dermatology, Sun Yat‐Sen Memorial Hospital Sun Yat‐sen University Guangzhou China

**Keywords:** ablative fractional CO₂ laser, atrophic scar, hypertrophic scar, scar treatment, treatment timing

## Abstract

**Background:**

Linear scars, resulting from surgical incisions or traumatic injuries, can pose both aesthetic and functional dilemmas. Ablative fractional CO₂ laser (AFCL) therapy has been recognized for its ability to enhance the appearance and flexibility of scars; however, the ideal timing for such treatments remains a subject of debate.

**Aims:**

This study retrospectively evaluates the effectiveness of AFCL in treating linear atrophic and hypertrophic scars, with a focus on identifying the optimal timing to achieve the best possible outcomes.

**Methods:**

Patients who underwent treatment for linear scars using AFCL at our hospital between January 2022 and July 2024 were included in the study. Participants were categorized into two groups: those with atrophic scars and those with hypertrophic scars. Hypertrophic scars were assessed using the Vancouver Scar Scale (VSS), whereas atrophic scars were evaluated with the Scar Cosmesis Assessment and Rating (SCAR) scale. Furthermore, considering the timing of scar formation—with a six‐month period as the threshold—two subgroups were categorized as early treatment and late treatment. The disparities in scar improvement rates were then computed and subjected to analysis.

**Results:**

Among 55 patients, 31 had atrophic scars and 24 had hypertrophic scars. AFCL treatment significantly improved clinical scores in both groups. The SCAR score for atrophic scars decreased from 6.50 (SD 1.31) to 4.92 (SD 1.71) (*p* < 0.001), and the VSS score for hypertrophic scars decreased from 6.02 (SD 0.46) to 2.73 (SD 0.39) (*p* < 0.001). The early‐treatment subgroup showed a 35.38% (SD 24.54%) improvement in atrophic scars, significantly higher than the 12.53% (SD 25.65%) in the late‐treatment subgroup (*p* = 0.018). No significant timing effect was found for hypertrophic scars (*p* = 0.764).

**Conclusion:**

AFCL is an effective treatment for linear scars. Early intervention, specifically within the first 6 months, leads to superior outcomes for atrophic scars. In contrast, the timing of treatment is less critical for hypertrophic scars.

**Trial Registration:**

Chinese clinical trial registry: Registration no. ChiCTR2400092038

## 1 Introduction

Scars are the end result of the wound healing process following skin injuries such as acne, burns, surgery, and trauma [1]. The clinical presentation of skin scars is diverse, and no consensus currently exists regarding their classification. Scars resulting from surgical and linear trauma are primarily classified as linear scars, which are further divided clinically into hypertrophic and atrophic scars based on their height relative to the skin surface [2]. The wound healing process generally comprises three stages: the inflammatory phase, the proliferative phase, and the remodeling phase. Abnormal collagen structure following the remodeling phase is the underlying cause of skin scar formation [1, 3].

Scars not only affect aesthetics but may also lead to pain, itching, restricted mobility, and a reduced quality of life for patients, making scar treatment a significant concern in dermatology. Treatment modalities for scars include topical applications, intralesional injections, physical therapies, laser and energy‐based techniques, and surgical interventions, among which laser therapy plays a crucial role [4, 5, 6]. Ablative fractional CO₂ laser (AFCL) has been proven effective in improving scar appearance, thickness, flexibility, and surface texture [7, 8]. By delivering high‐energy laser beams that create microthermal zones on the skin surface, AFCL induces photothermolysis, promotes rapid epithelial regeneration and dermal remodeling, and helps to disrupt abnormal scar tissue and release scar tension.

The optimal timing for CO₂ fractional laser treatment for scars remains a subject of considerable debate. This study retrospectively analyzes cases from a laser aesthetic center where CO₂ fractional laser was used to treat linear atrophic and hypertrophic scars, aiming to evaluate the optimal timing and therapeutic efficacy of CO₂ fractional laser in scar management.

## 2 Materials and Methods

### 2.1 Patients

This study included patients who received AFCL treatment for linear scars at the Laser Aesthetic Outpatient Clinic of the Dermatology Department, our hospital, Guangzhou, China, from January 2022 to July 2024. Inclusion criteria were as follows: (1) a confirmed diagnosis of linear scars and (2) complete clinical data. Exclusion criteria were as follows: (1) patients who received other scar treatments during the study period, (2) patients unable to comply with follow‐up after treatment, and (3) patients with keloid scars. Due to differences in evaluation criteria and efficacy between atrophic and hypertrophic scars, two experienced dermatologists classified patients into atrophic and hypertrophic scar groups for separate analysis. This study was approved by the Ethics Committee of our hospital (Approval No. SYSKY‐2024‐696‐01).

### 2.2 Treatment Procedure

Photographs of the scar area were taken before treatment. A topical anesthetic cream (compound lidocaine cream, Tongfang, China) was applied under occlusion with plastic film for 30 min. Prior to treatment, the area was cleansed with water, disinfected with benzalkonium bromide solution (our hospital, China), and then wiped dry with sterile cotton swabs. Patients were positioned supine, and an experienced operator used an AFCL (model JZ‐2, Guoxiong, China) to scan the scar area. Parameters were adjusted based on scar type and morphology, using either Manual mode or Deep mode. Energy settings were 1–3 W for Manual mode and 5–70 mJ with a spot spacing of 0.8–1.2 mm for Deep mode. For atrophic scars, Manual mode was used to perforate the scar center with 1.0–2.0 mm spacing to release adhesions, followed by deep mode for overall scar treatment with energy settings adjusted based on severity. For hypertrophic scars, manual mode was used to perforate the raised surface with 1.0–2.0 mm spacing to reduce scar volume, followed by Deep mode for comprehensive treatment with energy settings adjusted based on severity. The power settings for Deep mode were recorded and analyzed. Each session of fractional CO₂ laser therapy was performed with an interval of 1–3 months to allow adequate skin recovery. Patients were instructed to avoid sun exposure post‐treatment.

### 2.3 Scar Assessment Methods

Hypertrophic scars were assessed using the Vancouver Scar Scale (VSS) [9], which evaluates scar severity based on pigmentation, vascularity, pliability, and height, with a scoring range of 0–14. Atrophic scars were assessed using the Scar Cosmesis Assessment and Rating (SCAR) scale [10, 11], which evaluates scar spread, erythema, dyspigmentation, surgical or suture marks, hypertrophy/atrophy, and overall impression, with a scoring range of 0–13. Two experienced physicians independently assessed the scars using digital photographs taken before treatment and at least 1 month post‐treatment, with the average of their scores taken as the final score.

### 2.4 Statistical Analysis

All data were analyzed using IBM SPSS Statistics version 27.0 (IBM, USA). Qualitative data were described using frequencies, while quantitative data were described using range (minimum and maximum values), mean, standard deviation, and median. The Kolmogorov–Smirnov test was used to assess the normality of data distribution. For normally distributed data, paired *t*‐tests were used to compare pre‐ and post‐treatment scores. For non‐normally distributed data, the Wilcoxon signed‐rank test was applied. Additionally, in subgroup analyses, improvement rates of atrophic and hypertrophic scars were compared based on scar formation time (early treatment group: < 6 months; late treatment group: ≥ 6 months), calculated as: (post‐treatment score − pre‐treatment score)/pre‐treatment score. Independent sample *t*‐tests or Mann–Whitney *U* tests were used to further analyze score differences between subgroups, depending on data distribution. All statistical analyses were two‐tailed, with significance set at *p* < 0.05.

## 3 Result

This study included 55 patients with linear scars, comprising 31 patients with atrophic scars and 24 with hypertrophic scars. The clinical characteristics of the study population are shown in Table 1.

**TABLE 1:** Baseline of scar patients.

	Atrophic scar (*n* = 31)	Hypertrophic scar (*n* = 24)
Sex (M/F)	12/19	9/15
Age (mean ± SD, years)	25.1 ± 13.2	23.6 ± 14.2
Duration (median [$P_{25}$, $P_{75}$], years)	0.5 (0.1, 5.0)	0.4 (0.1, 1.0)
Sessions (median [min–max], times)	1 [1–6]	1 [1–6]
Power ([mean ± SD], MJ)	33.0 ± 15.0	25.3 ± 15.6
Early/late treatment	14/17	13/11
Location		
Face	28	16
Neck	2	4
Limb	1	4

### 3.1 Efficacy Analysis

Following AFCL treatment, clinical scores for both atrophic and hypertrophic scars showed significant improvement (Table 2). In the atrophic scar group, the mean SCAR score decreased from 6.50 (SD 1.31) before treatment to 4.92 (SD 1.71) post‐treatment, a statistically significant difference (*p* < 0.001). In the hypertrophic scar group, the mean VSS score decreased from 6.02 (SD 0.46) pre‐treatment to 2.73 (SD 0.39) post‐treatment, which was also statistically significant (*p* < 0.001).

**TABLE 2:** Impact of fractional CO_2_ laser treatment on different scars.

	SCAR or VSS (mean ± SD)		*t*	*p*
	Before treatment	After treatment		
Atrophic scar (*n* = 31)	6.50 ± 1.31	4.92 ± 1.71	4.81	< 0.001
Hypertrophic scar (*n* = 24)	6.02 ± 0.46	2.73 ± 0.39	6.92	< 0.001

### 3.2 Subgroup Analysis

To evaluate the effect of treatment timing on efficacy, patients were divided into early treatment (< 6 months) and late treatment (≥ 6 months) groups based on the duration of scar formation.

- Atrophic scars: 14 patients received early treatment, and 17 received late treatment. There were no significant differences between the two groups in terms of age, treatment energy, pre‐treatment SCAR score, or post‐treatment SCAR score. The average improvement rate in the early treatment group was 35.38% (SD 24.54%), significantly higher than the 12.53% (SD 25.65%) observed in the late treatment group (*p* = 0.018) (Table 3).
- Hypertrophic scars: 13 patients received early treatment, and 11 received late treatment. No significant difference in improvement rate was found between the two groups (*p* = 0.764) (Table 4).

**TABLE 3:** Comparison of immature and mature scars after treatment on atrophic scar.

	No. of patients	Age (mean ± SD, years)	Sessions (median [$P_{25}$, $P_{75}$])	Power (deep mode) (mean ± SD, MJ)	SCAR (mean ± SD)		Improvement rate (mean ± SD, %)
					Before	After	
< 6 months after injury	14	28.07 ± 14.18	1 [1, 2]	30.48 ± 17.89	6.54 ± 1.23	4.25 ± 1.89	35.38 ± 24.54
> 6 months after injury	17	22.64 ± 12.23	1 [1, 3]	35.07 ± 12.33	6.47 ± 1.41	5.47 ± 1.37	12.53 ± 25.65
*p* value	—	0.262	0.295	0.406	0.893	0.055	0.018

**TABLE 4:** Comparison of immature and mature scars after treatment on hypertrophic scar.

	No. of patients	Age (mean ± SD, yrs)	Sessions (median [$P_{25}$, $P_{75}$])	Power (deep mode) (mean ± SD, MJ)	VSS (mean ± SD)		Improvement rate (mean ± SD, %)
					Before	After	
< 6 months after injury	13	23.46 ± 15.77	1 [1, 1.5]	28.18 ± 15.37	7.03 ± 1.92	3.39 ± 2.17	50.28 ± 39.19
> 6 months after injury	11	23.82 ± 12.84	1 [1, 2]	21.85 ± 16.06	4.50 ± 1.53	1.96 ± 1.31	54.99 ± 36.32
*p* value	—	0.953	0.789	0.381	< 0.001	0.07	0.764

**FIGURE 1:**
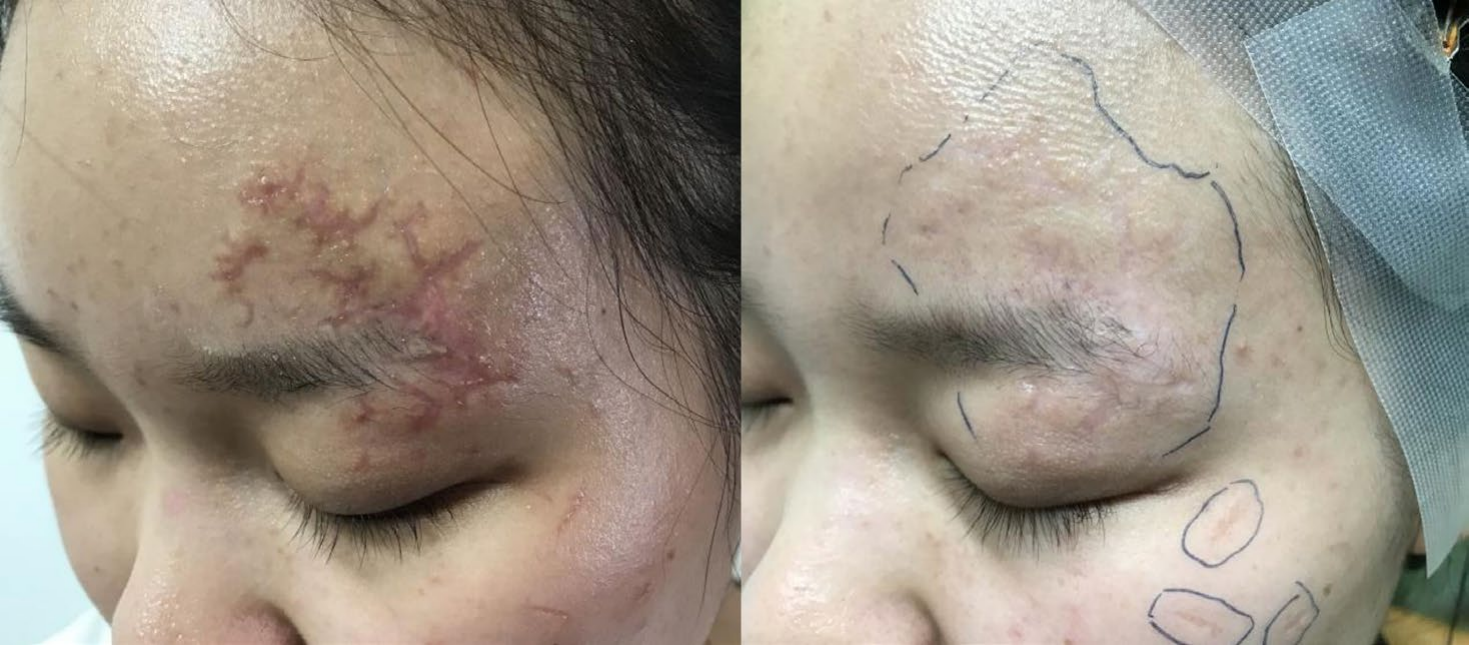
Atrophic scar at the early treatment group before and after treatment: 1 month post‐injury with a total of four sessions.

**FIGURE 2:**
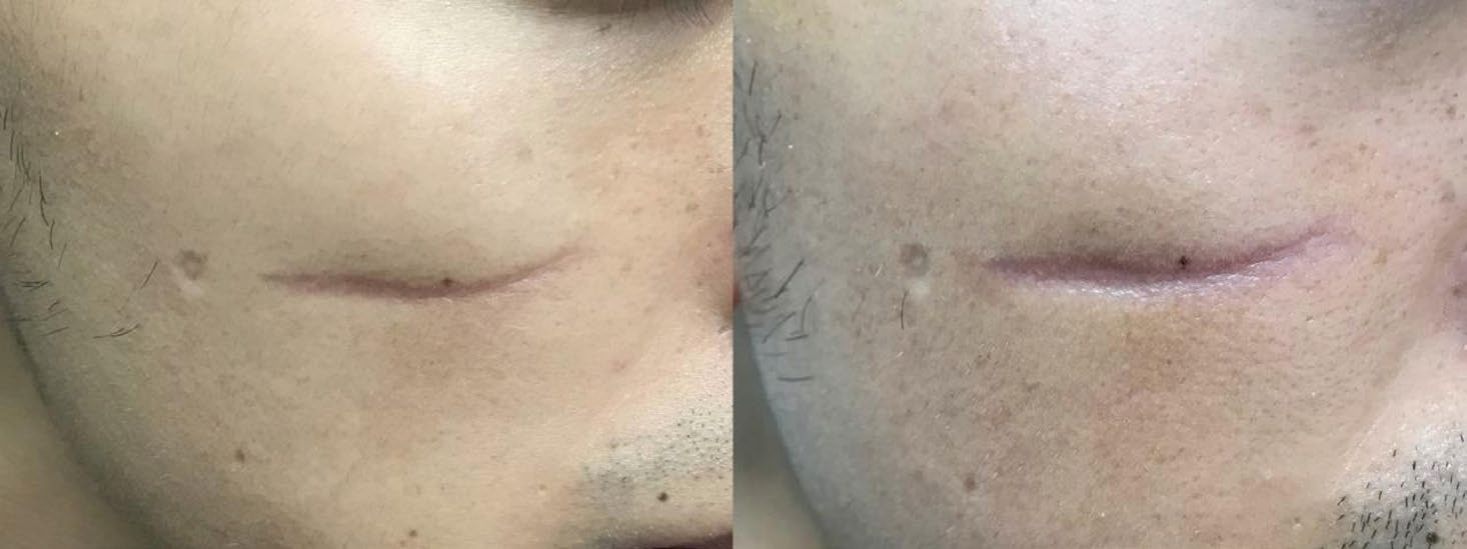
Atrophic scar in the late treatment group before and after treatment: 6 years post‐injury with a total of four sessions.

**FIGURE 3:**
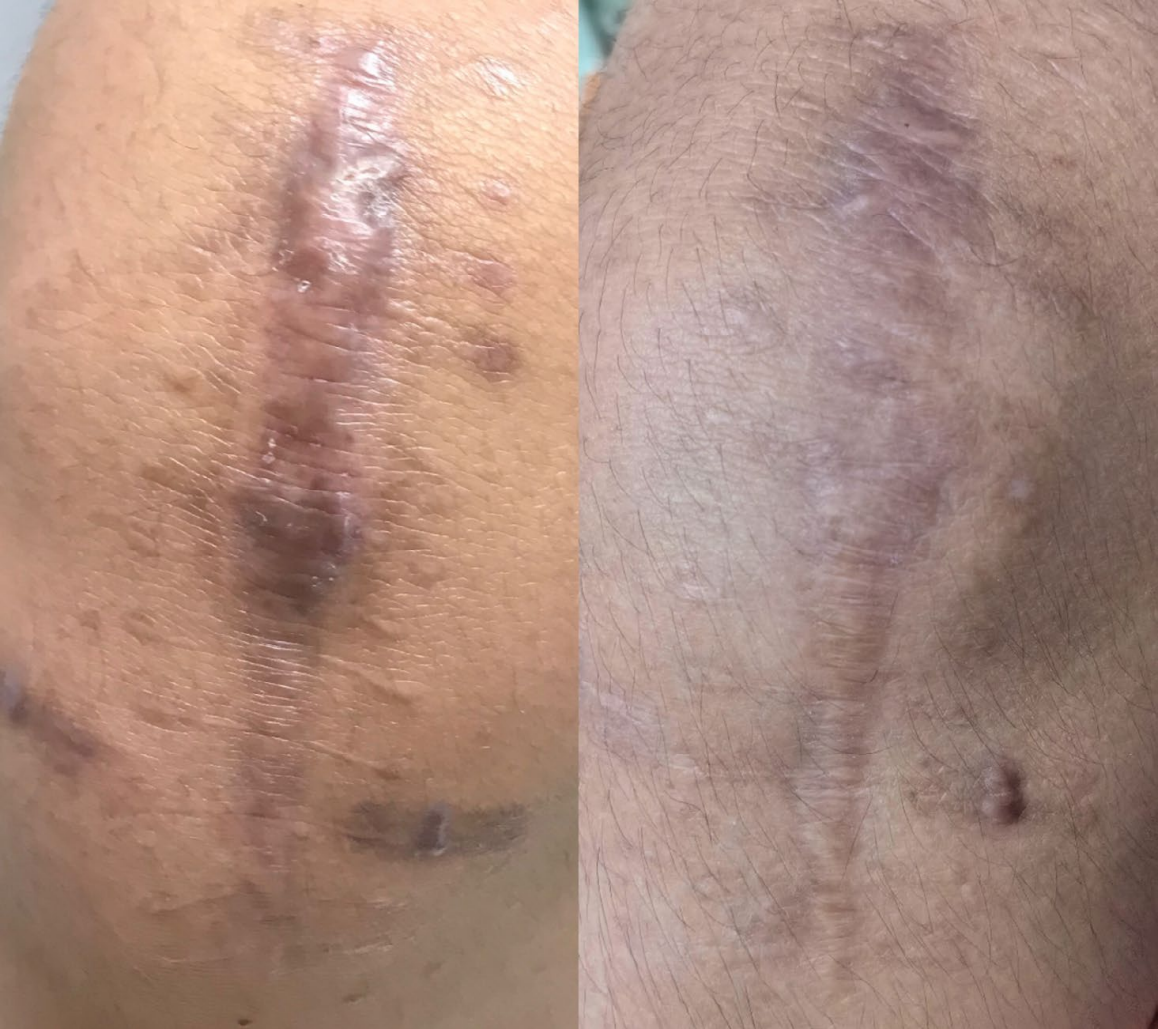
Hypertrophic scar at early treatment group before and after treatment: 5 months post‐injury with a total of one session.

**FIGURE 4:**
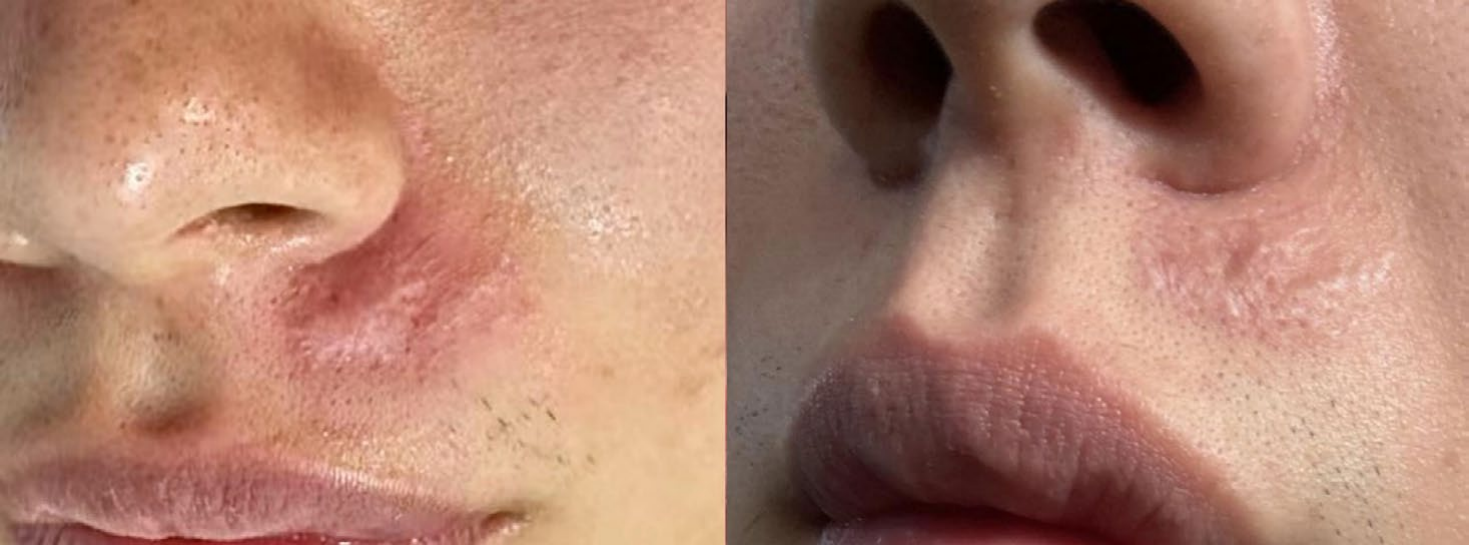
Hypertrophic scar at late treatment group before and after treatment: 6 years post‐injury with a total of three sessions.

Figures 1, 2, 3, 4 display representative cases from each subgroup.

## 4 Discussion

In this study, we retrospectively analyzed the efficacy of AFCL in treating different types of scars, with a particular focus on the impact of treatment timing on atrophic and hypertrophic scars. Consistent with previous literature, we found that AFCL significantly improved both hypertrophic and atrophic scars. However, our detailed analysis of treatment timing revealed efficacy differences between scar types.

### 4.1 Advantages of Early Treatment for Atrophic Scars

Current research on atrophic scars predominantly focuses on acne‐induced scars, with limited studies addressing atrophic scars from trauma and surgery. Weiss et al. [12] included 15 patients with non‐acne atrophic scars in their study and used a three‐dimensional optical imaging system (Primos Imaging) to quantitatively analyze treatment outcomes. After three sessions of AFCL treatment, significant improvements were observed in scar texture, pigmentation, atrophy, and overall appearance. Traditional clinical practice has favored treatment during the maturation or remodeling phase of scars, but recent studies increasingly emphasize the benefits of early intervention [13, 14, 15, 16]. Our study found that the efficacy of CO₂ fractional laser treatment for atrophic scars was closely related to the timing of laser intervention. Early treatment (< 6 months after scar formation) resulted in superior scar improvement compared to late intervention. Shen et al. [17] in a meta‐analysis highlighted that early laser treatment markedly improves pigmentation and vascular abnormalities in scars, making them more similar in appearance to normal skin. Furthermore, Karmisholt et al. [18] in a systematic review suggested that early laser intervention provides effective stimulation during the critical phase of collagen synthesis, promoting collagen regeneration and optimizing alignment, thereby enhancing scar texture and flexibility.

### 4.2 Minimal Impact of Treatment Timing on Hypertrophic Scars

In contrast to atrophic scars, the timing of CO₂ fractional laser intervention had a minimal impact on the efficacy of hypertrophic scar treatment. Our findings indicate that CO₂ fractional laser effectively improves hypertrophic scars regardless of whether it is applied during the early or late stages of scar formation. Shen et al. [17] noted that the efficacy of hypertrophic scar treatment is more dependent on laser energy density and spot size parameters. Most patients in our study received only a single fractional laser treatment (18 for atrophic scars and 18 for hypertrophic scars), yet achieved significant improvement, consistent with previous observations [17, 19].

The innovation of this study lies in elucidating the differences in efficacy based on treatment timing for different scar types with CO₂ fractional laser, particularly showing that early intervention significantly benefits atrophic scars more than late treatment. Although many studies have shown a clear advantage of early CO₂ fractional laser intervention for scars, we observed that this benefit is primarily confined to atrophic scars, with no significant difference in timing efficacy for hypertrophic scars. This difference may be due to the higher degree of fibrosis in hypertrophic scars, which might require less structural remodeling than atrophic scars.

### 4.3 Choice of Assessment Scales

For scar assessment, the VSS is widely used in hypertrophic scar studies, as it evaluates scar appearance in terms of pigmentation, vascularity, pliability, and height. However, VSS has limitations when assessing atrophic scars because it does not accurately reflect the degree of scar indentation. Therefore, this study used the SCAR scale, specifically designed for linear scars, to evaluate atrophic scars. The SCAR scale has demonstrated good reliability and validity in both live patient evaluations and high‐quality photographic assessments [11, 20].

### 4.4 Clinical Implications and Study Limitations

It is important to note that due to the prolonged natural course of scar maturation and individual variability, spontaneous improvement over time could influence short‐term treatment outcome evaluations. Karmisholt et al. reported that scar remodeling begins around 1 month post‐CO₂ fractional laser treatment, though substantial improvements may not become apparent until 6 months of follow‐up [21]. Therefore, future studies should consider longer follow‐up periods to more accurately assess the long‐term effects of CO₂ fractional laser treatment. Additionally, the sample size in our study was relatively small, and the follow‐up period was short, indicating the need for further studies with larger cohorts and extended follow‐up to enhance the generalizability and reliability of the results. This study did not include histopathological evaluation of collagen remodeling. Future prospective studies incorporating histological analysis will be crucial to validate and further understand the clinical results observed.

## Conflicts of Interest

The authors declare no conflicts of interest.

## Data Availability

The data that support the findings of this study are available from the corresponding author upon reasonable request.
